# Prognostic Implication of Predominant Histologic Subtypes of Lymph Node Metastases in Surgically Resected Lung Adenocarcinoma

**DOI:** 10.1155/2014/645681

**Published:** 2014-10-12

**Authors:** Kenichi Suda, Katsuaki Sato, Shigeki Shimizu, Kenji Tomizawa, Toshiki Takemoto, Takuya Iwasaki, Masahiro Sakaguchi, Tetsuya Mitsudomi

**Affiliations:** ^1^Division of Thoracic Surgery, Department of Surgery, Kinki University Faculty of Medicine, 377-2 Ohno-higashi, Osaka-sayama 589-8511, Japan; ^2^Division of Molecular Pathology, Department of Pathology, Hyogo College of Medicine, 1-3-6 Minatojima, Chuo-ku, Kobe 650-8530, Japan

## Abstract

The International Association for the Study of Lung Cancer, American Thoracic Society, and European Respiratory Society (IASLC/ATS/ERS) proposed a new classification for lung adenocarcinoma (AD) based on predominant histologic subtypes, such as lepidic, papillary, acinar, solid, and micropapillary; this system reportedly reflects well outcomes of patients with surgically resected lung AD. However, the prognostic implication of predominant histologic subtypes in lymph nodes metastases is unclear so far. In this study, we compared predominant subtypes between primary lung tumors and lymph node metastatic lesions in 24 patients with surgically treated lung adenocarcinoma with lymph node metastases. Additionally, we analyzed prognostic implications of these predominant histologic subtypes. We observed several discordance patterns between predominant subtypes in primary lung tumors and lymph node metastases. Concordance rates were 22%, 64%, and 100%, respectively, in papillary-, acinar-, and solid-predominant primary lung tumors. We observed that the predominant subtype in the primary lung tumor (HR 12.7, *P* = 0.037), but not that in lymph node metastases (HR 0.18, *P* = 0.13), determines outcomes in patients with surgically resected lung AD with lymph node metastases.

## 1. Introduction

Lung cancer is the leading cause of cancer-related mortality in the world [1], and lung adenocarcinoma (AD) is the most common histological type [2]. However, lung AD is not a uniform disease. Pathologically, lung AD includes several morphologic subtypes such as lepidic, papillary, acinar, micropapillary, solid, and mixture of these components. Because most lung ADs fall into the mixed subtype category [3], the International Association for the Study of Lung Cancer, American Thoracic Society, and European Respiratory Society (IASLC/ATS/ERS) recently proposed a new classification for lung AD based on the predominant histologic subtype [4]. The importance of this new AD classification, with wide applicability, is underscored by its prognostic effect [5–9]. Previous reports have strongly supported the role of the predominant histologic subtype of the “primary lung tumor” as a prognostic factor. However, predominant histologic subtypes often differ between primary lung tumors and their lymph node metastases [10]. Whether the predominant subtype of the primary lung tumor or of its lymph node metastases reflect prognosis better among patients with surgically resected lung AD with lymph node metastases is unclear. Therefore, we decided to compare the predominant histologic subtypes between primary lung tumors and their lymph node metastases and to analyze their value as prognostic factors.

## 2. Materials and Methods

### 2.1. Study Population

Patients who were diagnosed with lung AD from 2007 to 2008 were identified from the database of Division of Thoracic Surgery, Department of Surgery, Kinki University Faculty of Medicine. This database includes patients' clinicopathological information and survival data. Among 139 patients with pathologically proven lung AD, we included those with lymph node metastases in this study. Patients who underwent limited resections, wedge resection and segmentectomy, were excluded. None of the patients had neoadjuvant therapy prior to surgical resection.

### 2.2. Histologic Evaluation

Two investigators (Katsuaki Sato and Shigeki Shimizu), who were blinded to patients' clinical data, reviewed all hematoxylin and eosin-stained slides of the primary lung tumors. All cases were histologically evaluated according to the IASLC/ATS/ERS classification [4], with estimation in 5% increments of each histologic subtype present. All cases were classified based on the predominant histologic subtype. The lowest limit for the predominant subtype was set at 30% as previously described [11]. Metastatic lymph node lesions were evaluated by two investigators (Kenichi Suda and Shigeki Shimizu), who were blinded to patients' clinical data and primary lung tumor diagnoses. Histologic patterns present in the lymph nodes (both N1 and N2 lymph nodes, if applicable) were recorded as a binary variable as described previously [11]. Proportions of the two histologic subtypes were determined by one of the investigators (Shigeki Shimizu).

### 2.3. Statistical Analysis

Recurrence-free survival was defined as the time from pulmonary resection to the time that the patient survives without any signs or symptoms of cancer. Patients without an evidence of recurrence or a known date of death were censored at the time of the last follow-up. Overall survival was defined as the time from pulmonary resection to death. Patients without a known date of death were censored at the time of the last follow-up. Differences in recurrence-free and overall survival of the two groups were compared with the Kaplan-Meier method and Log-rank test. Multivariate analysis for overall survival was performed using the Cox proportional hazards modeling technique. These statistical analyses were performed with StatView version 5.01 (SAS Institute).

## 3. Results

### 3.1. Correlation of Predominant Histologic Subtypes between the Primary and Metastatic Sites

Our patient cohort included 24 patients of whom 12 were male (50%) and 15 were smokers (63%), with a median age of 62.5 years (range: 49–82 years). All were Japanese. Of the 24 patients, 13 had T1 disease (primary tumor size < 3.0 cm), and 14 had N2 lymph node metastases (Table 1). Predominant histologic subtypes in their primary lung tumors were 9 papillary-predominant, 11 acinar-predominant, and 4 solid-predominant nodules. None of our subjects had lepidic- or micropapillary-predominant primary lung tumors.

In the analyses of predominant subtype in lymph node metastases, the N1 and N2 lymph nodes were independently assessed. Among the 8 patients who developed both N1 and N2 lymph node metastases, predominant subtypes were the same in both lymph nodes in seven patients. The other one patient had acinar-predominant subtypes in his primary tumor and N1 lymph nodes, but developed a solid-predominant metastasis in his N2 lymph nodes. This patient was classified as having solid-predominant lymph node metastasis in the later analyses.

**Figure 1:**
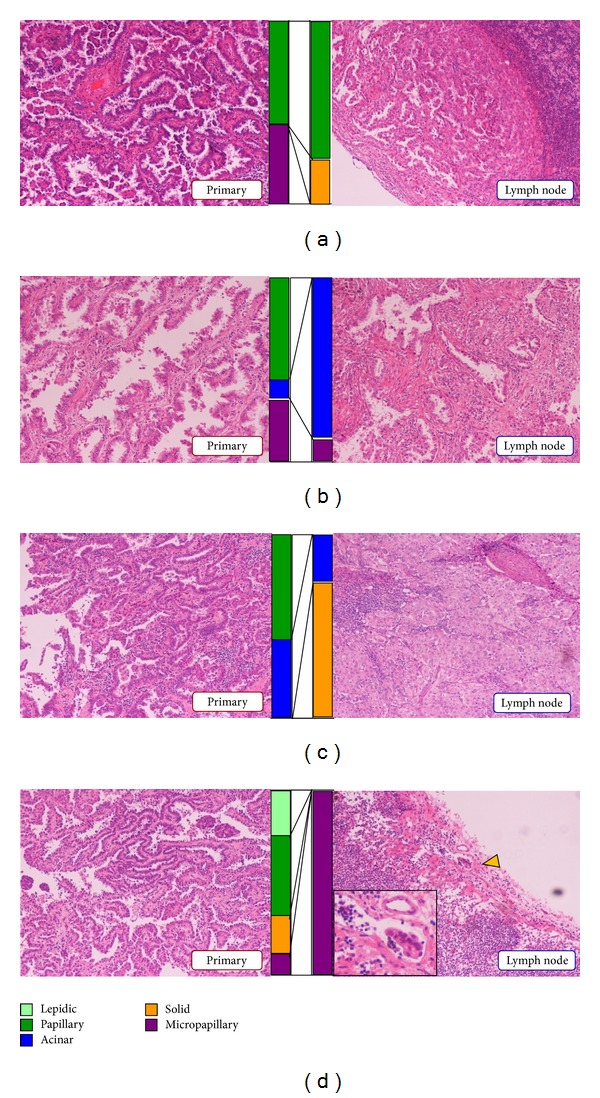
Representative photo-micrographs of primary lung tumors and metastatic lesions of lymph nodes. All of these four patients had papillary-predominant primary lung tumors (left), but developed lymph node metastases that were papillary-predominant (a), acinar-predominant (b), solid-predominant (c), and micropapillary-predominant (d), as shown on the right. Colored bar graphs indicate the proportion of histologic subtypes. In primary lung tumors (left), proportions of each histologic subtype present are estimated in 5% increments. Histologic subtypes in metastatic lymph nodes (right) were recorded as a binary variable.

**Figure 2:**
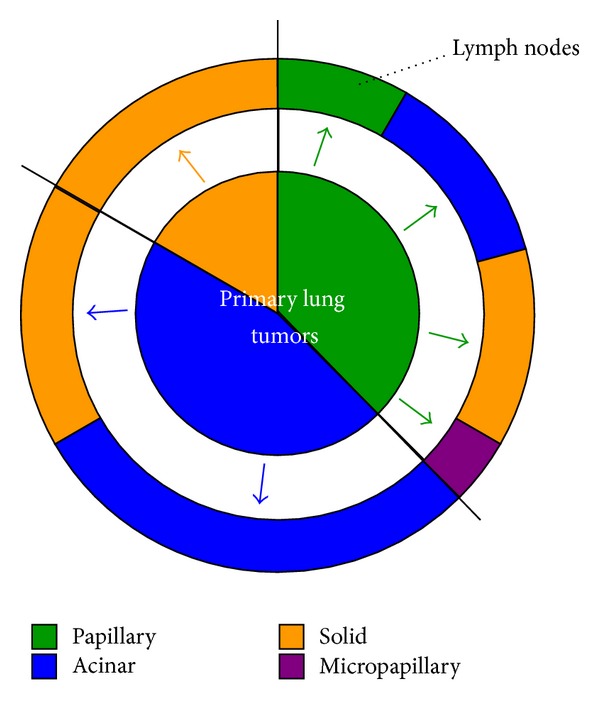
Correlation between predominant histologic subtype in primary lung tumors and that in lymph node metastases. Inside pie chart indicates primary lung tumors; outside chart indicates lymph nodes. Each color represents each predominant histologic subtype as shown below the pie charts.

In comparing predominant histologic subtypes between primary lung tumors and lymph node metastases, several discordant patterns were observed. Of 9 patients with papillary-predominant primary tumors, only 2 showed the same histology in their lymph node metastases; whereas the others had 3 acinar-, 3 solid- and 1 micropapillary-predominant metastases in their lymph nodes (Figures 1 and 2). In 11 patients with acinar-predominant primary lung tumors, 7 retained the same histology in metastatic lymph nodes, while the other 4 patients had solid-predominant node metastases (Figure 2). However, all 4 patients with solid-predominant primary lung tumors developed node metastases with the same predominant subtype (Figure 2).

### 3.2. Prognostic Implication of Predominant Histologic Subtypes in Primary and Metastatic Sites

**Figure 3:**
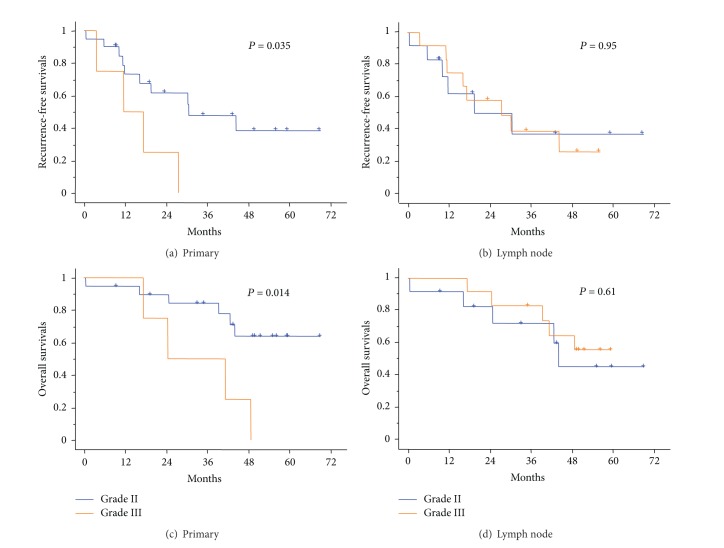
Kaplan-Meier survival curves for recurrence-free survival and overall survival stratified according to the predominant histologic subtypes of primary lung tumors (a and c) and metastatic lymph nodes (b and d). Grade II includes papillary/acinar-predominant lesions and Grade III includes solid/micropapillary-predominant lesions, according to the previous report [10].

Next, we analyzed the prognostic implication of these predominant histologic subtypes on recurrence-free survival and overall survival. In these analyses, papillary- and acinar-predominant tumors were grouped in Grade II, whereas solid- and micropapillary-predominant tumors were grouped in Grade III, following the previous report [10]. Lung AD of Grade I, lepidic-predominant lesions, was not present in our cohort. In univariate analyses, patients with Grade III primary lung tumors had a significant poor prognosis (*P* = 0.014; Figures 3(a) and 3(c)). However, predominant histologic subtype in node metastases was not predictive of prognosis (Figures 3(b) and 3(d)). In multivariate analysis for overall survival adjusted for age, primary tumor size, and N2 lymph node involvement (Table 2), Grade III subtype in the primary tumors still significantly predicted poor prognosis (Hazard ratio: 12.7; *P* = 0.037), but predominant histologic subtype in the lymph nodes did not (*P* = 0.13). In addition, among patients with Grade II primary lung tumor, predominant histologic subtype in node metastases was not predictive of recurrence-free survival (*P* = 0.71) and overall survival (*P* = 0.38).

## 4. Discussion

At present, pathologic stage is the most important indicator for adjuvant chemotherapy after pulmonary resection for lung AD. However, treatment outcomes of adjuvant chemotherapies are not satisfactory, so far. For example, platinum-doublet adjuvant chemotherapy only improves the 5-year survival rate by 5.4% compared with surgery alone [12]. This means that only one out of 20 patients actually benefits from adjuvant chemotherapy. To concentrate patients who will benefit from adjuvant chemotherapy, or to identify patients with higher risk of recurrence, numerous biomarkers that may predict patients' outcomes have been reported. Among several approaches for this point, the IASLC/ATS/ERS lung AD classification has several benefits. This approach can be performed with standard histologic techniques and incorporated into routine anatomic diagnosis of the excised tumor without the need to acquire and process additional tissue [10]. The usefulness of the IASLC/ATS/ERS lung AD classification as a prognostic factor has been reported in several large-scale retrospective analyses [5–7].

However, whether histologic subtype in the primary lung tumor or that in the lymph node metastases more accurately predicts outcomes for patients with surgically resected lung AD with lymph node metastases is unclear. Relationships of predominant histologic subtypes between primary lung tumors and metastatic lymph nodes have been only scantly reported. Sica et al. reported in their analysis of 73 patients that concordance between predominant histology in primary lung AD tumors and their metastases (lymph node or brain) was 100% for micropapillary, 86% for solid, 42% for acinar, and 23% for papillary types of AD [10]. In their analysis, concordance was defined as “…the predominant pattern observed in the primary tumor is present at the metastatic site.” Russell et al. observed that the concordance of the predominant histologic subtype in primary tumor and N2 lymph node metastases was 100% for micropapillary, 92% for solid, 42% for acinar, and 0% for papillary [11]; they defined concordance as “…the predominant subtype in the primary tumor is present and most prominent in the corresponding N2 metastases.” In the present study, discordance patterns of each predominant histologic subtype were similar to these previous observations.

To our knowledge, there has been only one previous study of prognostic implications of predominant histologic subtype in metastatic lymph nodes. That study of 69 mainly Caucasian patients found no significant differences based on nodal predominant subtypes, which indicated that the predominant subtype in the primary tumor was the main determinant of outcome [11]. In this study, we have obtained similar results, using a different cohort with different ethnicity. Therefore, our results, together with the previous ones, indicate that in-depth histologic evaluation of only primary tumors but not for node metastases is necessary to predict outcomes for patients with surgically resected lung AD with lymph node metastases.

## 5. Conclusions

In this study, we observed several patterns among discordant predominant histologic subtypes between the primary lung tumors and their lymph node metastasis in surgically resected lung ADs. Additionally, we found that the predominant subtype in the primary lung tumor, but not that of the lymph node metastases, determines outcomes in patients with lung adenocarcinoma with lymph node metastases.

## Figures and Tables

**Table 1:** Patients characteristics.

Characteristics	
Gender	
Female/male	12/12
Age	
Median (range)	62.5 (49–82)
Smoking status	
Ever/never	15/9
Tumor size	
T1/T2–4	13/11
Lymph node status	
N1/N2	10/14
Predominant subtype in primary tumor	
Papillary/acinar/solid	9/11/4

**Table 2:** Univariate and multivariate analysis for overall survival.

Characteristics	Univariate analysis		Multivariate analysis		*P* value
	HR	95% CI	HR	95% CI	
Age group					
Under 62 years/over 63 years	2.4	0.67–8.57	1.5	0.30–7.25	0.64
Tumor size					
T2–4/T1	0.76	0.21–2.69	0.85	0.20–3.57	0.83
Lymph node metastasis					
N2/N1	1.9	0.49–7.46	0.81	0.15–4.37	0.80
Predominant subtype in primary tumors					
Grade III/Grade II	4.4	1.21–15.9	12.7	1.17–142.9	0.037
Predominant subtype in lymph nodes					
Grade III/Grade II	0.72	0.21–2.51	0.18	0.02–1.66	0.13

HR: hazard ratio; 95% CI: 95% confidence interval; Grade II includes papillary- and acinar-predominant tumors and Grade III includes micropapillary- and solid-predominant tumors.
